# Connectome-based biophysical models of pathological protein spreading in neurodegenerative diseases

**DOI:** 10.1371/journal.pcbi.1012743

**Published:** 2025-01-21

**Authors:** Peng Ren, Xuehua Cui, Xia Liang

**Affiliations:** 1 Research Center for Social Computing and Information Retrieval, Harbin Institute of Technology, Harbin, China; 2 Institute of Science and Technology for Brain-Inspired Intelligence and Department of Neurology, Huashan Hospital, Fudan University, Shanghai, China; 3 Frontiers Science Center for Matter Behave in Space Environment, Harbin Institute of Technology, Harbin, China; Inria, FRANCE

## Abstract

Neurodegenerative diseases are a group of disorders characterized by progressive degeneration or death of neurons. The complexity of clinical symptoms and irreversibility of disease progression significantly affects individual lives, leading to premature mortality. The prevalence of neurodegenerative diseases keeps increasing, yet the specific pathogenic mechanisms remain incompletely understood and effective treatment strategies are lacking. In recent years, convergent experimental evidence supports the “prion-like transmission” assumption that abnormal proteins induce misfolding of normal proteins, and these misfolded proteins propagate throughout the neural networks to cause neuronal death. To elucidate this dynamic process in vivo from a computational perspective, researchers have proposed three connectome-based biophysical models to simulate the spread of pathological proteins: the Network Diffusion Model, the Epidemic Spreading Model, and the agent-based Susceptible-Infectious-Removed model. These models have demonstrated promising predictive capabilities. This review focuses on the explanations of their fundamental principles and applications. Then, we compare the strengths and weaknesses of the models. Building upon this foundation, we introduce new directions for model optimization and propose a unified framework for the evaluation of connectome-based biophysical models. We expect that this review could lower the entry barrier for researchers in this field, accelerate model optimization, and thereby advance the clinical translation of connectome-based biophysical models.

## Introduction

Neurodegenerative diseases are disorders primarily characterized by the progressive degeneration and death of neurons. The nervous system is progressively impaired by these diseases, resulting in severe disruptions of cognitive and motor functions. Typically, these diseases are caused by the aggregation and accumulation of abnormal proteins within neurons, leading to neuronal dysfunction and cell death. However, the pathogenic mechanisms remain incompletely understood.

In various neurodegenerative diseases, normally folded proteins undergo misfolding under specific circumstances, becoming infectious pathological proteins. Pathological proteins further induce misfolding of normal proteins into new pathological proteins. The brain region where pathological proteins first appear is defined as the seed region, from where pathological proteins spread along the brain structure network to the entire brain. This “prion-like transmission” behavior ultimately leads to the degeneration of neurons and pathological changes in brain tissue [1]. The pathological transmission mechanisms of neurodegenerative diseases such as Alzheimer’s disease (AD), Parkinson’s disease (PD), frontotemporal dementia (FTD), amyotrophic lateral sclerosis, and other neurodegenerative diseases are similar to those of prions [1,2]. However, evidence from studies on human patients to support this hypothesis remains quite limited.

In recent years, the development of neuroimaging technology has provided unprecedented opportunities for noninvasive characterization of pathological protein accumulation and structural/functional abnormalities in neurodegenerative diseases, as well as for examining the hypothesis of “prion-like transmission.” For example, structural MRI can quantitatively depict the volume, shape, and position of brain regions; PET can visually display the distribution of pathological proteins in various brain regions following injection of radiolabeled tracers [3]; functional MRI can obtain brain activity signals without damage. Moreover, diffusion-weighted MRI can noninvasively characterize the connectivity patterns (connectome) between different brain regions throughout the brain. The advancement of these noninvasive brain imaging technologies has provided convenience for revealing the potential pathological propagation mechanisms of neurodegenerative diseases.

By analyzing neuroimaging data from patients through statistical methods, a pioneering study conducted by Seeley and colleagues [4] evaluated the role of network connectivity in neurodegenerative diseases, including AD, behavioral variant frontotemporal dementia (bvFTD), semantic dementia (SD), progressive nonfluent aphasia (PNFA), and corticobasal syndrome (CBS). They discovered that the syndromic atrophy foci anchor large-scale functional networks in the healthy brain. Moreover, mounting evidence has shown that adjacent brain regions that are structurally connected to the seed region, rather than simply being physically nearby, exhibit higher levels of structural changes in numerous brain disorders [5]. Specifically, studies on Alzheimer’s disease have revealed that the regions that are structurally connected to, rather than in close physical proximity to the putative seed region (medial temporal lobe) for the transmission of tau pathological proteins in AD, exhibit a close correlation between the degree of connectivity to seed region and the distribution of tau protein [6–10]. In other words, these findings indicate that brain regions with stronger connections to the seed region are likely to be more susceptible to disease. Therefore, researchers have proposed that pathological proteins are produced from one or more brain regions (seed regions) and propagate through the brain connectome to the entire brain. Although it remains to be proven directly in humans, current research suggests that brain regions with the highest levels of pathological protein accumulation and neurodegeneration often tend to be those that are highly connected to the seed region [4,11–14]. Altogether, this evidence supports the hypothesis that pathological proteins propagate across neurons from a qualitative perspective.

In order to further quantitatively characterize the development process of neurodegenerative diseases, researchers extended the above qualitative evidence by establishing connectome-based biophysical models, which can quantify the dynamic changes of pathological protein distribution over time. In 2012, Raj and colleagues [14] established the first connectome-based biophysical model characterizing the propagation of pathological protein across neuronal connections, known as the Network Diffusion Model (NDM). NDM simulates the spread of pathological proteins based on the concentration gradient of pathological proteins between brain regions, which is constrained by the structural connectome of the human brain. In 2014, Iturria-Medina and colleagues [15] proposed the Epidemic Spreading Model (ESM). Compared to NDM, ESM takes the immune brain response against abnormal protein accumulation [16–19] into account and describes the relationship between the production, clearance, propagation of pathological protein and disease progression. In 2019, Zheng and colleagues [18] proposed that both of the aforementioned concentration-based simple diffusion models follow a first-order kinetics law [14,15], which is mathematically easy to solve but has limited explanatory power for pathology. Therefore, Zheng and colleagues introduced an agent-based Susceptible-Infected-Removed (SIR) model [18]. The 3 propagation models have provided essential research tools and enabled a better understanding of the propagation processes and mechanisms of different pathological proteins within the brain. In turn, this review contributes to elucidating the impact of pathological propagation on brain structure and function, as well as provides theoretical foundations and experimental evidence for the diagnosis, treatment, and prevention of diseases. Here, we will start by introducing the fundamental principles of the 3 propagation models, after which we will review their applications in various neurodegenerative diseases. Then, we will summarize the strengths and weaknesses of these models, offering guidance to researchers in selecting the appropriate model. Finally, we will propose a universal evaluation framework for connectome-based biophysical models, which standardizes the entire process from data management and model selection to model evaluation.

## Biophysical models of pathological protein spreading

Previous studies have indicated that the propagation of pathological protein in AD and other neurodegenerative diseases typically follows the neural networks. However, there is a lack of direct evidence supporting this viewpoint. While many studies found supporting evidence based on the spatial distribution of pathological proteins and patterns of lesions, these observations only provide correlational evidence and do not directly demonstrate the processes and mechanisms of protein propagation. In this context, Raj and colleagues [14] introduced the NDM in 2012. Subsequently, the ESM and agent-based Susceptible-Infectious-Removed model were proposed to study the spreading of pathological protein quantitatively.

## Network diffusion model

In 2012, Raj and colleagues [14] established the NDM for the first time, using functions to characterize the dynamic propagation of pathological proteins quantitatively. The NDM primarily addresses 2 questions: (1) What mechanisms does the prion-like spreading follow? (2) What are the macroscopic consequences of the propagation of pathological protein? The NDM takes all pathological proteins as pathogenic factors, and the diffusion of these pathogenic factors is restricted to the fiber pathways defined by the brain’s structural connectivity network. The neuronal degeneration and macroscopic brain atrophy processes are simulated through trans-synaptic spreading, driven by concentration differences of pathogenic factors between different neurons.

### Mathematical principles

The NDM models the progression of neurodegenerative diseases as the diffusion process of misfolded proteins in the brain network *G*={*V*,*E*}, where node *v*_*i*_∈*V* denotes the *i*th region of interest (ROI_i_), and edge *e*_*i*,*j*_∈*E* denote the fiber pathways connecting ROI_i_ and ROI_j_ [14]. Infected brain regions are defined as ROI_2_ and uninfected brain regions are defined as ROI_1._ The misfolded proteins (MP) spread from ROI_2_ to ROI_1_, with the number of infected cells being modeled as the product of the MP concentration *x*_2_ at ROI_2_ and the connection strength *c*_1,2_ between the 2 ROIs. Simultaneously, there is reverse diffusion from ROI_1_ to ROI_2_, denoted as *x*_1_*c*_1,2_. Therefore, within a short time interval *δt*, the increase in the total MP concentration at ROI_1_ is *β*(*x*_2_−*x*_1_)*c*_1,2_*δt*, where *β* is a diffusion coefficient constant controlling the propagation speed. Assuming bidirectional diffusion, as *δt*→0, a first-order linear differential equation can be derived:

$$\frac{dx_{1}}{dt}=\beta c_{1,2}(x_{2}-x_{1}).$$ (1)

Introducing knowledge from graph theory, the model analogizes the diffusion of MPs between nodes to the entire network. Assuming that the concentration of MP at each node in the network at time *t* is represented by the vector ***x***(*t*) = {*x*(*v*,*t*),*v*∈*V*}, then the generalized version of Formula 1 on the whole network is:

$$\frac{dx(t)}{dt}=-\beta Hx(t).$$ (2)

Where *H* is the weighted Laplacian matrix that is defined as the difference between the degree matrix *D*_*IJ*_ and the weighted adjacency matrix *A*_*IJ*_, the diagonal element *D*_*II*_ characterizes the degree of each node *I*, and the element of *A*_*IJ*_ represents the ratio of mean fiber number *n*_*IJ*_ and length *l*_*IJ*_ between nodes *I* and *J*.


$$\begin{array}{l} \;H_{IJ}=D_{IJ}-A_{IJ}\;\mathrm{with}A_{IJ}=\frac{n_{IJ}}{l_{IJ}}\\ \mathrm{and}\;D_{II}=\mathrm{diag}\sum_{J=1,J\neq I}^{N}A_{IJ} \end{array}\}$$ (3)

***x***(*t*) is modeled as:

$$x(t)=e^{-\beta Ht}x_{0}.$$ (4)

***x***_0_ is the MP concentration vector of each node at baseline, *e*^−*βHt*^ essentially acts as a spatial and temporal blurring operator, and this equation is referred to as the diffusion kernel.

Assuming that the atrophy at ROI_k_ is a result of MP accumulation, the atrophy can be modeled as Formula 5:

$$\varphi_{k}(t)=\int_{0}^{t}x_{k}(\tau )d\tau .$$ (5)

In the entire brain network, it is modeled as follows:

$$\varphi (t)=\int_{0}^{t}x(\tau )d\tau .$$ (6)

### Applications in diseases

Raj and colleagues [14] first validated whether NDM can predict the development process of neurodegenerative diseases. They applied NDM to simulate the behavior of MP propagation on whole-brain structural connectivity derived from tractography data of 14 healthy subjects. They then computed the eigenmodes of the NDM to determine if the large-scale patterns of disease observed in various types of dementia, such as normal aging, AD, and bvFTD, were consistent with the network-based diffusion patterns. The results of the NDM analysis showed that specific eigenmodes represented unique spatial patterns that resemble the known atrophy patterns of different types of dementia. Subsequently, to investigate whether the NDM model aids in clinical diagnosis, Raj and colleagues [19] conducted longitudinal follow-up prediction studies. Researchers first used NDM to predict brain atrophy and metabolic change patterns at the end of the follow-up study based on the baseline brain atrophy patterns of 418 patients. The study results showed a significant correlation between real data and simulated data (brain atrophy, *R* = 0.85; metabolism, *R* = 0.75). Furthermore, researchers examined 2 local growth models, namely, the sigmoid model [20] and the exponential model [21], for the same prediction task. The results indicated that the performance of these 2 models was significantly worse than that of NDM.

In addition to its applications in AD, NDM has also been widely used in other neurodegenerative diseases. Table 1 lists examples of NDM applications. In 2019, Freeze and colleagues [22] used NDM to predict the pathological protein spreading of PD and explored whether the brain regional transcription of PD risk genes could predict the seed regions of PD pathological protein spreading. They first simulated the propagation using different ROIs as seed points. The correlation coefficient *R* between predicted and observed values was taken as the likelihood of a brain region being the seed region for pathological protein spreading. The results showed that the substantia nigra had the highest *R* and was the most likely seed region (*R*^*2*^ = 0.65). Subsequently, researchers found significant differences in the expression of PD risk genes, as well as a correlation between the expression of PD risk genes and the seed region inferred by NDM. The transcriptional pattern of *HLA-DQA1* and *HLA-DRB6* genes was identified as significant predictors of seed regions, and these genes were also highly expressed in the substantia nigra. These results suggest that brain regional gene expression can predict the seed regions of PD pathological protein spreading. Pandya and colleagues [23] investigated the possibility of applying NDM to a rare neurodegenerative disease, progressive supranuclear palsy (PSP). Using different brain regions as seed points, they simulated the propagation on an undirected human brain connectivity network and calculated the correlation coefficient *R* between predicted and observed values at various time points. By comparing the *R* in different brain regions, they found that the hypothalamus had the highest *R* and was likely the seed region for PSP pathological protein spreading (*R* = 0.42). Subsequently, researchers repeated the above simulation with anterograde and retrograde directional connectivity and found a significantly increased correlation coefficient, reaching 0.48 and 0.53, respectively. The result indicates that directed propagation forms of human brain connectivity better fit the real data. NDM has many applications in other diseases as well. For example, Pandya and colleagues [24] used NDM to simulate the pathological protein spreading of amyotrophic lateral sclerosis (ALS), providing a better understanding of the disease’s mechanisms. Additionally, Parsons and colleagues [25] used NDM to evaluate the spread of small neurological events in coronavirus disease, helping to reveal the virus’s spreading pathways and impact on the nervous system. Similarly, Poudel and colleagues [26] used NDM to predict the progression of brain lesions in traumatic brain injury patients, providing a basis for prediction and clinical intervention. Furthermore, Kuceyeski and colleagues [27] employed NDM to predict recovery from coma in patients with severe brain injury, providing insights for intervention and treatment.

**Table 1:** Application of NDM in neurodegenerative diseases.

Literatures	Diseases	Subjects	Simulation data	Connectome	Seed	Correlation coefficient
Raj and colleagues [14]	AD, bvFTD	14 HC, 18 AD, 18 bvFTD	Cross-sectional T1w MRI	HARDI	-	AD: *R* = 0.23, bvFTD: *R* = 0.27
Raj and colleagues [19]	AD	168 HC, 99 AD, 151 MCI	Cross-sectional T1w MRI, Tau-PET scans	DTI	-	Atrophy: *R* = 0.85, Metabolism: *R* = 0.75
Torok and colleagues [28]	AD	175 HC, 117 AD,156 LMCI, 148 EMCI	Longitudinal T1w MRI, Tau-PET scans	DTI	HIP, L, HIP, R, Amy, R	*R* = 0.76
Freeze and colleagues [22]	PD	117 HC, 232 PD	Cross-sectional T1w MRI, AHBA	HARDI	SN	*R*^*2*^ = 0.65
Pandya and colleagues [23]	PSP	40 HC, 150 PSP	Longitudinal T1w MRI	DTI	HT	Normal: *R* = 0.42, Anterograde: *R* = 0.48, Retrograde: *R* = 0.53
Pandya and colleagues [24]	ALS	79 ALS	Longitudinal T1w MRI	DTI	Insula	*R* = 0.45
Parsons and colleagues [25]	COVID-19	123 COVID-19	Cross-sectional T1w MRI	HARDI	Bilateral CB	*R* = 0.51
Poudel and colleagues [26*]*	TBI	19 HC, 17 TBI	Longitudinal T1w MRI	HARDI	-	*R* = 0.56
Freeze and colleagues [29]	sCJD	85 sCJD	Cross-sectional T1w MRI	HARDI	PCC	*R* = 0.53
Poudel and colleagues [30]	HD	26 HC, 26 HD	Cross-sectional T1w MRI	DWI	-	*R* = 0.69

The *R* in the table are all Pearson’s coefficients and represent the maximum values during the experimental process. The column “Simulation Data” and “Connectome” in the table respectively denote the data modalities utilized in the experiment and the data modalities employed for connectome generation.

AD, Alzheimer’s disease; bvFTD, behavioral variant frontotemporal dementia; HC, healthy control; T1w MRI, T1-weighted MRI; HARDI, high angular resolution diffusion imaging; MCI, mild cognitive impairment; DTI, diffusion tensor imaging; LMCI, late MCI; EMCI, early MCI; HIP, hippocampi; L, left; R, right; Amy, amygdala; PD, Parkinson’s disease; AHBA, Allen human brain atlas; SN, substantia nigra; PSP, progressive supranuclear palsy; HT, hypothalamus; ALS, amyotrophic lateral sclerosis; CB, cerebellum; COVID-19, Corona Virus Disease 2019; TBI, traumatic brain injuries; sCJD, sporadic Creutzfeldt–Jakob disease; PCC, posterior cingulate cortex; HD, Huntington’s disease; DWI, diffusion weighted imaging.

In order to improve model performance, researchers often combine NDMs with other mathematical models to predict the pathological protein and atrophy spreading in neurodegenerative diseases. Detailed literature can be found in Table 2. Common methods and applications of combined models are listed below:

1. Combining NDM with kinetic models. Fornari and colleagues [31] proposed to combine local kinetic models and NDM to capture both protein misfolding and spreading of the prion-like pathological propagation process. Specifically, in ascending order of complexity, the one-concentration Fisher–Kolmogorov model and the two-concentration Heterodimer model could be combined with the NDM. The Fisher–Kolmogorov model has a single unknown parameter of the misfolded protein concentration c, which converts healthy protein to misfolded one at a rate *α*. The Heterodimer model has 2 unknown parameters: the healthy concentration *p* and misfolded concentration $\tilde{p}$. This model produces healthy proteins at a rate *k*_0_, clears healthy and misfolded proteins at rates *k*_1_ and $\tilde{k_{1}}$, and converts healthy proteins to misfolded ones at a rate *k*_12_; these parameters collectively represent the processes of recruitment, misfolding, and fragmentation. Moreover, Fornari and colleagues [32] proposed a nucleation-aggregation-fragmentation extension of NDM dynamics that incorporates the theory of aggregation using the Smoluchowski model, which has *n* unknown parameters *c*_*i*_, one for the concentration of each size *i* = 1,…,n, where *c*_1_ represents the concentration of healthy monomers. The model produces healthy proteins at a rate *k*_0_, clears healthy and misfolded proteins at rates *k*_1_ and *k*_*i*_, nucleates 2 misfolded proteins from 2 healthy proteins at a rate *k*, aggregates by adding single healthy proteins to misfolded filaments at a rate *a*, and fragments misfolded filaments at a rate *f*. They found that the modeling of A*β* and tau on the connectome of healthy participants showed different size distributions and propagation along the network, as well as various stages with different dynamics and invasive properties. Similarly, Raj and colleagues [33] also encapsulated the process of AD tau protein production, aggregation, and diffusion throughout the entire brain in a single model. They used the Smoluchowski equation [34] to model tau aggregation and NDM to model tau propagation, creating a new aggregation-network-diffusion (AND) model. By seeding in the entorhinal cortex, the simulation with the AND model accurately captured the progression of tau protein throughout the brain and reproduced the brain atrophy of AD.

2. Using discrete Smoluchowski’s equations to model A*β* and tau interaction. Bertsch and colleagues [35] proposed a comprehensive and flexible extension of NDM dynamics with the description of the evolution and interplay between A*β* and tau proteins plus the generation of amyloid plaques. Specifically, the model was constructed to simulate the diffuse of tau protein on the connectivity graph (i.e., neural network) and the diffuse of A*β* protein in the proximity graph (i.e., extracellular space), as well as their interactions. The model controls respectively the A*β* agglomeration (*α*), the production of monomeric tau driven by A*β* oligomers (*C*_*τ*_), and the tau seeding at the entorhinal cortex (*c*). By constructing the model on healthy connectome using different combinations of parameters, they examined the amyloid cascade hypothesis, the source of tau monomers (A*β* oligomers or tau seeding at EC) and also the drug effect on agglomeration of A*β*.

3. Using reaction-diffusion models on graphs as a unified modeling framework. Vergni and colleagues [36] introduced flexible reaction-diffusion models that are implemented on the connectome, which overcome the strong constraint of conserved substances in simple NDMs. The reaction-diffusion models incorporate the reaction of the substance inside nodes and allow various reaction terms that are suitable for different neurodegenerative diseases. By fixing other parameters, they showed how different reaction terms (logistic growth term, neutral Allee effect, and the strong Allee effect) and initial conditions (concentration and seed position) can influence the evolution dynamics on the healthy connectome, especially compared to that of simple NDM.

4. Using NDM output as predictors in linear regression models. Damasceno and colleagues [37] used baseline patterns of tau and Aβ level in participants as independent variables and established 4 linear regression models (m_1_, m_2_, m_3_, and m_4_) using different weights and combinations methods. Subsequently, researchers modeled the longitudinal change of tau protein within the time interval *Δ*t as the product of *m*, the Laplace operator *H* in Eq 3, and baseline tau protein level ${\vec{\tau }}_{BL}$, i.e., $\Delta \vec{\tau }=mH{\vec{\tau }}_{BL}\Delta t$, which is simply a discrete version of Eq 2. To predict future tau protein burden, Damasceno and colleagues [37] conducted an 18-month follow-up study on 60 mild cognitive impairment (MCI) patients and 28 AD patients, collecting longitudinal PET scan data for each patient. They used the model mentioned above to predict the longitudinal changes in tau protein levels within target brain regions. The results showed that the model accurately predicted the spatial distribution of future tau protein based on the spatial pattern of baseline tau protein. There was a significant correlation between real data and predicted data (MCI: *R*^*2*^ = 0.65 ± 0.16, AD: *R*^*2*^ = 0.71 ± 0.11). Similarly, Acosta and colleagues [38] selected different AD-related genes and NDM-predicted atrophy or metabolism as independent variables of multiple linear regression models. To explore the contribution of vulnerable genes and structural connectivity to the linear model, researchers first performed simulations with each brain region as the seed region separately. The results showed that simulated atrophy using the entorhinal cortex as the seed region yielded the highest correlation coefficient with real atrophy data. Then, using the entorhinal cortex as the seed region, researchers employed different linear models for prediction. These linear models included (1) using NDM-predicted atrophy or metabolism as the sole independent variable in the linear model; and (2) choosing any one gene from the selected group of genes as the sole independent variable in the linear model. The Pearson’s correlation coefficients between real data and simulated data are *R*^*2*^ = 0.34 and *R*^*2*^∈[0.014, 0.16], respectively. This indicated that NDM could better explain AD pathology relative to genes. By using NDM-predicted atrophy or metabolism and any one gene as independent variables in the linear model, the model performed significantly better compared to models that only used genes as independent variables ($R_{max}^{2}$ = 0.44), further emphasizing the importance of connectome in the propagation of AD pathological protein.

5. Combining NDM with sparsity constraint methods. Torok and colleagues [28] employed NDM and regularization methods to construct a reverse inference algorithm, which infers the seed region of pathological protein spreading in AD and MCI patients. They first used *L*_2_ regularization constraint to find the earliest time point where there was the highest correlation between real baseline atrophy and NDM-predicted atrophy patterns. Subsequently, a cost function was established to measure the correlation between NDM-predicted atrophy patterns and baseline atrophy patterns at that time point. To ensure the sparsity of the seed regions, researchers added a *L*_2_ regularization term to the cost function, restricting the seed points to common brain regions such as the hippocampus, entorhinal cortex, amygdala, and locus coeruleus. The 2 processes mentioned above were further improved by adding weight coefficients. At the group level, by randomly combining seed regions and using longitudinal MRI data, the new model identified the seed regions that yielded the highest correlation between real atrophy and simulated atrophy while keeping the seed points sparse: the left/right hippocampal regions and the right amygdala. At the individual level, researchers compared the simulation of longitudinal atrophy patterns using 2 types of seed regions: personalized seed regions predicted by the model and the common hippocampal seed region. The results showed that the personalized seed regions performed better than the hippocampal seed region, which has important implications for individualized disease risk assessment and treatment planning for AD patients. Similarly, Yang and colleagues [39] limited the number or locations of tau seed regions by introducing source terms *s*(*t*) on the right-hand side of the NDM equation. Subsequently, researchers used gradient descent to constrain the seed regions to as few brain regions as possible. To characterize the tau distribution in preclinical AD, the research team obtained tau-PET images for 62 normal aging brains at time points (*t*_1_,*t*_2_,*t*_3_) and performed prediction using the models mentioned above. They predicted tau distribution at *t*_2_ based on the tau distribution at *t*_1_ and predicted tau distribution at *t*_3_ based on the tau distribution at *t*_2_. The results showed that the peak fits between the predicted distribution and observed distribution were *R*^*2*^ = 0.88 and *R*^*2*^ = 0.62, respectively. Moreover, they identified several brain regions with a high possibility of being seed regions, including the inferior temporal lobe, fusiform gyrus, entorhinal cortex, and parahippocampal gyrus. All the studies mentioned above consistently demonstrate the potential of NDM to be applied in neurodegenerative diseases.

## Epidemic spreading model

The previous part introduces the NDM [14] that utilizes concentration differences between different brain regions as the driving force for propagation, quantitatively analyzing the spatiotemporal changes in pathological proteins. However, NDM has 2 limitations. Firstly, NDM models the diffusion process between brain regions as propagation between nodes, but one ROI can consist of multiple neurons, NDM does not consider the deposition and propagation of MP within ROIs [40,41]. Secondly, there is no production and clearance term in NDM for MP and only diffusion of MP between neighboring ROIs was set in the way that preserves the amount of MP, which cannot be satisfactory in the case of a progressive disease. To better characterize the progression of neurodegenerative diseases, it is essential to introduce the production and clearance terms. Studies have shown that the brain’s immune response can slow down the accumulation of MP [42–45]. For example, macrophages and microglial cells can clear Aβ, thereby reducing their deposition to some extent [46–48]. Moreover, clearance can also occur via enzymatic degradation (e.g., for amyloid beta) [49], physical clearance from the brain (e.g., via the glymphatic system) [50], or the lysosomal system (e.g., for alpha-synuclein) [51]. Considering the interaction between the brain’s clearing system and pathological protein, the ESM proposed by Iturria-Medina and colleagues [15] addresses the limitations of NDM. Firstly, ESM models the infection of ROIs as 2 parts: endogenous infection and exogenous infection. Endogenous infection refers to the spread of MP from infected neurons to uninfected neurons within ROIs (microscopic scale). When endogenous infection gets severe, it manifests as the deposition of MP. Exogenous infection refers to the spread of MP between infected brain regions and uninfected brain regions (macroscopic scale). Secondly, ESM considers the clearance process of MP in the brain. Infected brain regions have a certain probability of initiating a clearance process to eliminate pathological substances and protect the brain region.

**Table 2:** The extended models of NDM.

Literatures	Subjects	Simulation	Connectome	Models	Seed	Correlation
Fornari and colleagues [31]	418 HC from HCP	-	DTI	NDM+ kinetic models	Different seed was set for different research questions	The concentration dynamics on healthy connectome was qualitatively compared to previous reports, no quantitative comparison was made
Fornari and colleagues [32]	418 HC from HCP		DTI	NDM+ Smoluchowski equations		
Bertsch and colleagues [35]	Budapest Reference Connectome:		DTI	NDM+ Smoluchowski equations		
Vergni and colleagues [36]	B.A.T.M.A.N.:		HARDI	Reaction-diffusion models		
Raj and colleagues [33]	**ADNI:** 117 AD, 156 LMCI, 148 EMCI **Yonsei University:** 53 AD, 52 aMCI, 23 naMCI, 67 HC	Cross-sectional T1w MRI, Tau-PET scans	DTI	NDM + Smoluchowski equations	EC	*R* = 0.637
Damasceno and colleagues [37]	60 MCI, 28 AD	Longitudinal T1w MRI, Tau-PET scans	DTI	Graph Laplacian + baseline tau protein + susceptibility	EC	*R*^*2*^ = 0.65 ± 0.16
Acosta and colleagues [38]	153 AD, 327 EMCI, 2 MCI, 201 LMCI, 232 HC	Cross-sectional T1w MRI, AHBA, FDG-PET scans	HARDI	NDM + linear models	EC	*R*^*2*^ = 0.44
Hu and colleagues [52]	**Dataset1**: 188 AD, 400 MCI, 229 HC **Dataset2**: 98 AD, 207 MCI, 163 HC	Cross-sectional T1w MRI, Longitudinal T1w MRI	DTI	NDM + sparse impulsive stimulations	-	Maximum mean square errors = 2.46
Yang and colleagues [39]	62 subjects from Harvard Aging Brain Study	Longitudinal T1w MRI, Tau-PET scans	DTI	NDM + sparse source localization	-	*R*^*2*^ = 0.88

The *R* in the table are all Pearson coefficients and represent the maximum values during the experimental process. The column “Simulation Data” and “Connectome” in the table respectively denote the data modalities utilized in the experiment and the data modalities employed for connectome generation.

ADNI, Alzheimer’s disease neuroimaging initiative database; AD, Alzheimer’s disease; MCI, mild cognitive impairment; LMCI, late MCI; EMCI, early MCI; aMCI, amnestic MCI; naMCI, non-amnestic MCI; HC, healthy control; MRI, magnetic resonance imaging; T1w MRI, T1-weighted MRI; DTI, diffusion tensor imaging; NDM, network diffusion model; EC, entorhinal cortex; AHBA, Allen human brain atlas; HARDI, high angular resolution diffusion imaging.

### Mathematical principles

The brain is viewed as a network composed of nodes (ROIs) and edges (white matter fibers) [15], and the distribution of abnormal proteins in the brain can be characterized using probability distribution functions of PET imaging. Here, *P*_*i*_ represents the probability that ROI_i_ is infected. ESM simulates the propagation of MP in the brain network by using a structural connectivity matrix and a vector consisting of the probability of MP deposition in each ROI.

The dynamic changes in ESM depend on the interactions between infected and uninfected brain regions. The infection rate of ROI_i_ over time can be modeled as a nonlinear differential equation:

$$\frac{dP_{i}}{dt}=(1-P_{i}(t))\varepsilon_{i}(t)-\delta_{i}(t)P_{i}(t)+ℵ.$$ (7)

The first term (1−*P*_*i*_(*t*))*ε*_*i*_(*t*) on the right-hand side of the equation represents the production of the possibility 1−*P*_*i*_(*t*) that ROI_i_ is not infected and the probability *ε*_*i*_(*t*) that it might be infected by MP. The second term *δ*_*i*_(*t*)*P*_*i*_(*t*) represents the probability *P*_*i*_(*t*) that ROI_i_ is already infected and the probability *δ*_*i*_(*t*) that MP in this brain region may be cleared. The third term ℵ represents the error induced by noise, which follows a Gaussian distribution, but the parameters μ and σ are unknown.

Here, *ε*_*i*_(*t*) represents the probability of uninfected ROIs being infected. Since each ROI is composed of multiple neurons, the infection rate is modeled as 2 parts: endogenous infection and exogenous infection, as represented by Eq 8.


$$\varepsilon_{i}(t)=\underset{j\neq i}{\sum }Pa_{j\to i}\beta_{j}^{ext}(t-\tau_{ij})P_{j}(t-\tau_{ij})+Pa_{i\to i}\beta_{i}^{int}(t)P_{i}(t).$$ (8)

On the right-hand side of Eq 8, the first term $\sum_{j\neq i}Pa_{j\to i}\beta_{j}^{ext}(t-\tau_{ij})P_{j}(t-\tau_{ij})$ represents the probability of exogenous infection. *Pa*_*j*→*i*_ represents the probability of structural connectivity network from ROI_j_ to ROI_i_, $\beta_{j}^{ext}(t-\tau_{ij})$ represents the external infection rate at time *t*−*τ*_*ij*_, *τ*_*ij*_ is the time MP takes to travel from ROI_i_ to ROI_j_, with a propagation rate of *V*_*MP*_ [53]. The second term $Pa_{i\to i}\beta_{i}^{int}(t)P_{i}(t)$ represents endogenous infection. Exogenous propagation means the spreading of MP across brain regions, while endogenous infection represents MP deposition within ROI.

Assuming the total infection rate is equal to the sum of endogenous infection rate and exogenous infection rate, i.e., $\beta_{i}(t)=\beta_{i}^{int}(t)+\beta_{i}^{ext}(t)$, the proportion of each rate is distinguished using the *Gini* coefficient. Therefore, an adjustment function *g*(*t*) (*Gini* coefficient) is introduced:

$$\beta_{i}^{ext}(t)=g(t)\beta_{i}(t)$$ (9)

$$\beta_{i}^{int}(t)=(1-g(t))\beta_{i}(t).$$ (10)

The *β*_*i*_(*t*) in Eq 9 can be modeled with *P*_*i*_ and constant parameter *β*_0_:

$$\beta_{i}(t)=\beta_{i}(P_{i},\beta_{0})=1-e^{-\beta_{0}P_{i}(t)}.$$ (11)

From Eqs 9–11, it can be observed that the higher $\beta_{i}^{int}(t)$ is, the more MP deposition occurs, making it easier to become seed regions for pathological protein spreading. The higher $\beta_{i}^{ext}(t)$ is, the stronger the MP’s ability to propagate, making it more likely to infect other brain regions.

Back to Eq 7, in the second term on the right-hand side, *δ*_*i*_(*t*) represents the probability of MP clearance, which can be modeled with *P*_*i*_(*t*) and a constant parameter *δ*_0_. In actual pathological progression, as the accumulation of MP deposition within a brain region, the clearance capacity of neurons decreases exponentially. Therefore, the clearance rate is modeled using an exponential function.


$$\delta_{i}(t)=\delta_{i}(P_{i},\delta_{0})=e^{-\delta_{0}P_{i}(t)}.$$ (12)

In conclusion, there are 4 unknown parameters in ESM: *δ*_0_, *β*_0_, *μ*, and *σ* that directly determine the dynamic evolution of the system.

### Applications in diseases

ESM has been widely applied to simulate the pathological protein spreading of AD. Researchers have utilized ESM to investigate relationships between different factors in AD. For example, do Aβ and tau proteins interact with each other? Is the clearance of pathological protein by the human brain’s immune system correlated with the onset age of AD? What roles do genes play in promoting the development of AD? Many studies have indicated that ESM can successfully reproduce the process of pathological protein propagation in neurodegenerative disease, providing an effective tool for unraveling the mechanisms of pathological protein spreading. Table 3 lists the literature relevant to ESM simulations of AD pathological protein propagation. In 2021, Iturria-Medina and colleagues [54] further optimized ESM and encapsulated the code into a cross-platform software, enabling the representation of multi-scale and multi-factor neuropathologic mechanisms (www.neuropm-lab.com).

**Table 3:** Application of ESM in AD.

Literatures	Subjects	Simulation data	Connectome	Seed	Correlation
Iturria-Medina and colleagues [15]	193 HC, 233 EMCI, 196 LMCI, 111 AD	Cross-sectional T1w MRI	DSI	-	*R*^*2*^ = 0.46 ~ 0.56
Vogel and colleagues [8]	162 HC, 89 MCI, 61 AD	Cross-sectional T1w MRI, Aβ-PET scans, Tau-PET scans	DTI	EC	*R*^*2*^ = 0.78
Jacob and colleagues [55]	1,143 Subjects	Cross-sectional T1w MRI	DTI	EC, MT, FFG, IT	*R*^*2*^ = 0.704, *R*^*2*^ = 0.266, *R*^*2*^ = 0.592, *R*^*2*^ = 0.503
Levitis and colleagues [56]	**DIAN**: 249 Subjects **ADNI**: 737 Subjects **OASIS**: 510 Subjects	Cross-sectional T1w MRI, Aβ-PET scans, Tau-PET scans	DSI	PCC, caudal ACC	*R*^*2*^ = 0.51

The *R* in the table are all Pearson coefficients and represent the maximum values during the simulation process. The column “Simulation Data” and “Connectome” in the table respectively denote the data modalities utilized in the experiment and the data modalities employed for connectome generation.

HC, healthy control; MCI, mild cognitive impairment; EMCI, early MCI; LMCI, late MCI; AD, Alzheimer’s disease; T1w MRI, T1-weighted MRI; DSI, diffusion spectrum imaging; DTI, diffusion tensor imaging; EC, entorhinal cortex; MT, middle temporal; FFG, fusiform gyrus; IT, inferior temporal; DIAN, dominantly inherited Alzheimer’s network database; ADNI, Alzheimer’s disease neuroimaging initiative database; OASIS, open access series of imaging studies; PCC, posterior cingulate cortex; ACC, anterior cingulate cortex.

Ituria-Medina and colleagues [15] were the first to use ESM to predict the propagation process of Aβ in AD. They conducted the following research: (1) obtained Aβ PET scan data of the subjects and calculated the Aβ deposition probability for each ROI, representing the possibility of Aβ deposition occurring in the ROI; (2) selected 6 brain regions that may experience Aβ deposition in the early stages of AD and used a random combination of these 6 brain regions as seed regions; (3) established a connectome using diffusion spectrum imaging (DSI) data from 60 healthy subjects; and (4) for each participant, the researchers set the probability of Aβ deposition in different seed regions and set the simulation time to 50 years. The distribution pattern of Aβ in the connectome was recorded on a 1-day basis. By continuously adjusting the parameters of the ESM through iterative algorithms, the correlation coefficient between the simulated and real Aβ distribution pattern was maximized, thereby obtaining 4 model parameters (generation rate *β*_0_, clearance rate *δ*_0_, noise level *μ* and *σ*) and the brain regions where Aβ first appeared. They examined 733 participants and ultimately found that the ESM simulated data could explain 46% to 56% variance of real Aβ deposition patterns in the brain, successfully reproducing the real pattern of Aβ deposition.

For pathological tau proteins, Vogel and colleagues [8] used ESM to simulate tau spreading with tau-PET data from 312 AD subjects and found that ESM could explain 70% of the variance in real data. To validate the close relationship between Aβ and tau proteins, Iturria-Medina and colleagues [15] examined the relationship between levels of Aβ^1–42^, total tau, and hyperphosphorylated tau in cerebral spinal fluid from different disease severity groups. The results revealed that Aβ production and clearance rates significantly influenced the total tau protein levels in cerebral spinal fluid, explaining 4.45% of the variance (*P* = 1.68 × 10^−5^). By analyzing the relationship between real Aβ deposition pattern and simulated pattern of tau protein, Vogel and colleagues [8] found that observed tau levels were higher than the predicted level in regions with high Aβ deposition. Altogether, these 2 studies suggest that Aβ plays a facilitating role in the propagation of tau during AD progression.

To explore whether the spatiotemporal patterns of Aβ proteins are consistent between autosomal dominant AD (ADAD) and sporadic AD, Levitis and colleagues [56] used ESM to reconstruct Aβ spatiotemporal patterns in both AD subtypes. Most ADAD and sporadic AD cases showed similar Aβ spatiotemporal patterns, with seed regions overlapping with the default mode network. However, 13% of the ADAD subjects had seed regions located in the striatum [53].

The classic Braak staging systematically describes the propagation pattern of tau protein at the tissue level as AD progression. However, clinical practice has observed heterogeneous individual spatiotemporal patterns of tau protein propagation [57,58]. To investigate differences in pathological protein spreading between AD subtypes, Vogel and colleagues [55] used tau-PET imaging data from 1,612 subjects and the Subtype and Stage Inference (SuStaIn) model to establish the spectrum of AD. The SuStaIn model selected 30 spatiotemporal features, where time features represented disease severity, and spatial features included 30 classic ROIs. The SuStaIn model determined the spatiotemporal trajectories of tau protein for 4 AD subtypes: limbic-predominant (severe pathology in the limbic system and medial temporal lobe), medial temporal lobe (MTL)-sparing (severe pathology in the occipital and parietal lobes), posterior temporal (early pathological tau deposition in the occipital lobe), and lateral temporal (left hemisphere pathology more severe than right hemisphere) [55]; 88% of subjects could be categorized into one of these 4 subtypes. The 2 follow-up visits for 513 subjects revealed that 88.5% of subjects were classified as the same AD subtype in both follow-up visits. Finally, the researchers identified seed regions for each of the 4 subtypes: entorhinal cortex, middle temporal gyrus, fusiform gyrus, and inferior temporal gyrus. In summary, Vogel and colleagues [55] demonstrated that ESM could characterize the propagation process of different subtypes, which has important implications for the individualized diagnosis and treatment of AD.

## Agent-based susceptible-infectious-removed model

In both NDM and ESM models, the propagation rate of pathological proteins between brain regions is influenced by concentration gradients, and the spreading speed obeys the law of mass effect with first-order kinetics [14,15]. Such models are mathematically easy to solve but have limited explanatory power. Instead, Zheng and colleagues [18] turned their attention to another ESM: the agent-based Susceptible-Infectious-Removed (SIR) model. We will explain the basic principles of SIR and its applications in neurodegenerative diseases.

### Mathematical principles

The SIR model is commonly used in the epidemiology study to simulate the propagation of infectious diseases within human populations [59]. In the SIR model, the population is divided into 3 groups: susceptible, who are not currently infected but are vulnerable to contracting the disease; infected, who are currently carrying the disease and can spread it to those in the susceptible group; and removed, who have been infected and are no longer able to spread the disease, either through recovery with immunity, isolation, or death [18]. When adapting the SIR model to simulate the pathological transmission of neurodegenerative diseases, the 3 groups are redefined as follows: susceptible individuals represent normal proteins that have not yet been affected, infected individuals represent misfolded proteins that have already been infected, and removed individuals represent cleared proteins, including both normal and misfolded proteins.


$$\alpha_{i}=\emptyset_{0,1}(gene\;expression_{i})$$ (13)

#### 1. The production rate of agent

Assuming that gene expression, which refers to the local expression of the gene encoding the relevant protein implicated in the production process, follows a normal distribution, ∅_0,1_(*gene expression*_*i*_) is the cumulative distribution function of the gene expression distribution, which represents the production rate of agents. The higher ∅_0,1_ means the higher the production rate of agents. After a duration of Δ*t*, the increment of agents is *α*_*i*_*S*_*i*_Δ*t*, where *S*_*i*_ is the number of voxels (volume) of ROI_i_.

#### 2. The clearance rate of normal and misfolded agent

The normal agents (NA) and misfolded agents (MA) are always in a dynamic process of production and clearance. Assuming the clearance rate is *β*_*i*_:

$$\beta_{i}=\emptyset_{0,1}(gene\;expression_{i})$$ (14)

Assuming that gene expression, which refers to the local expression of the gene encoding the relevant protein implicated in the clearance process, follows a normal distribution, ∅_0,1_(*gene expression*_*i*_) is the cumulative distribution function of the gene expression distribution, representing the clearance rate of the agent. Assuming that the proportion of NA and MA that remain active after a duration Δ*t* is $lim_{\delta \tau \to 0}{\left(1-\beta \delta \tau \right)}^{\frac{\Delta t}{\delta \tau }}=e^{-\beta \Delta t}$, the cleared proportion within the duration Δ*t* is 1−*e*^−*βΔt*^. As Δ*t* increases, the proportion of cleared agents also increases.

#### 3. Infectious process

Eq 15 represents the probability of NA, which has not been cleared after Δ*t*, becoming infected:

$$\gamma_{i}=1-e^{M_{i}\ln(1-\gamma_{0}^{i})}$$ (15)

Here, *M*_*i*_ is the number of MA, $\gamma_{0}^{I}$ represents the probability that NA becomes infected by MA at ROI_i_, denoted as $\frac{1}{S_{i}}$. ${\left(1-\gamma_{0}^{i}\right)}^{M_{i}}$ is the probability that NA has not been infected by any MA. Similarly, with a duration of Δt, the probability of NA not being infected is $lim_{\delta \tau \to 0}{\left(1-\gamma_{0}^{i}\delta \tau \right)}^{\frac{M_{i}\Delta t}{\delta \tau }}=e^{-\gamma_{0}^{i}M_{i}\Delta t}$, the probability of NA being infected by MA is $1-e^{-\gamma_{0}^{i}M_{i}\Delta t}$.

So, after a duration of Δt, the increment in NA is:

$$\Delta N_{i}=\alpha_{i}S_{i}\Delta t-(1-e^{-\beta_{i}\Delta t})N_{i}.$$ (16)

When the system reaches equilibrium, both NA and MA exist, and the increments follow Formulas 17 and 18:

$$\Delta N_{i}=\alpha_{i}S_{i}\Delta t-(1-e^{-\beta_{i}\Delta t})N_{i}-(e^{-\beta_{i}\Delta t})(1-e^{-\gamma_{0}^{i}M_{i}\Delta t})N_{i}$$ (17)

$$\Delta M_{i}=(e^{-\beta_{i}\Delta t})(1-e^{-\gamma_{0}^{i}M_{i}\Delta t})N_{i}-(1-e^{-\beta_{i}\Delta t})M_{i}.$$ (18)

#### 4. The propagation of agent

The agents could propagate between neurons within ROIs and can also propagate from ROI to an edge, where an edge represents a white matter tract connecting 2 ROIs. Formulas 19 and 20 represent the probability that the agent accumulates within ROI_i_ and the probability that it propagates to edge(i,j):

$$P_{regioni\to regioni}=\rho_{i}$$ (19)

$$P_{regioni\to edge(i,j)}=(1-\rho_{i})\frac{\omega_{ij}}{\sum_{j}\omega_{ij}}.$$ (20)

Where *ω*_*ij*_ is the connectivity strength of edge(i,j). Formulas 21 and 22 represent the probability that the agent at the edge(i,j) spreads to ROI_j_ and the probability that it stay in edge(i,j):

$$P_{edge(i,j)\to regionj}=\frac{1}{l_{ij}}$$ (21)

$$P_{edge(i,j)\to edge(i,j)}=1-\frac{1}{l_{ij}}.$$ (22)

N_ji_ and M_ji_ denote the concentration of NA and MA at the edge(i,j), after a duration of Δ*t*, the increment of NA and MA at ROI_i_ could be modeled with Formulas 23 and 24:

$$\Delta N_{i}=\underset{j}{\sum }\frac{1}{l_{ji}}N_{ji}\Delta t-(1-\rho_{i})N_{i}\Delta t$$ (23)

$$\Delta M_{i}=\underset{j}{\sum }\frac{1}{l_{ji}}M_{ji}\Delta t-(1-\rho_{i})M_{i}\Delta t.$$ (24)

The increment of NA and MA in the edge(i,j) could be represented with Formulas 25 and 26:

$$\Delta N_{ij}=(1-\rho_{i})\frac{\omega_{ij}}{\sum_{j}\omega_{ij}}N_{i}\Delta t-\frac{1}{l_{ij}}N_{ij}\Delta t$$ (25)

$$\Delta M_{ij}=(1-\rho_{i})\frac{\omega_{ij}}{\sum_{j}\omega_{ij}}M_{i}\Delta t-\frac{1}{l_{ij}}M_{ij}\Delta t.$$ (26)

The SIR model models brain atrophy as the result of 2 processes: (1) increasing toxicity within ROI leads to neuron death; (2) neuron death occurring in adjacent ROI causes the loss of projection neurons, resulting in deafferentation and atrophy in target regions. Brain atrophy over an interval Δ*t* can be modeled using Formula 27:

$$\Delta L_{i}(t)=k_{1}(1-e^{-r_{i}(t)\Delta t}+k_{2}\sum_{j}\frac{w_{ji}}{\sum_{j}w_{ji}}(1-e^{-r_{j}(t-1)\Delta t}).$$ (27)

Here, *r*_*i*_(*t*) is the proportion of MA in ROI_i_ at time point *t*, $1-e^{-r_{i}(t)\Delta t}$ represents the atrophy of ROI_i_ caused by accumulation of MA after a duration of Δ*t*, $1-e^{-r_{j}(t-1)\Delta t}$ represents the atrophy in ROI_i_ caused by neuronal death in adjacent ROI_j_ at time point *t*−1. The 2 coefficients *k*_1_ and *k*_2_ follows that *k*_1_+*k*_2_ = 1. According to experiments conducted by Zheng and colleagues [18], the model performance remains robust when the value of $\frac{k_{1}}{k_{2}}$ falls within the range of 0.1 to 10.

### Applications in diseases

The SIR models are widely used in multiple neurodegenerative diseases. Table 4 lists examples of SIR applications. In 2019, Zheng and colleagues [18] first applied the SIR model to the spreading of pathological protein in PD. They used cross-sectional T1w structural MRI data and generalized q-sampling imaging (GQI) data to characterize the real atrophy patterns and brain connectome, respectively. The SIR model was used to simulate the process of pathological α-synuclein (α-syn) proteins spreading throughout the brain connectome. The primary works included: (1) They used SIR to calculate the concentration of normal α-syn in each brain region of healthy individuals in a balanced state. (2) They used the cumulative distribution function of gene expression of *SNCA* (the gene encoding α-syn) and *GBA* (the gene encoding glucocerebrosidase) to represent the production and clearance rates of α-syn. (3) The concentration of normal proteins in the balanced state was used as the initial state of the system, and the concentration of misfolded α-syn (MP) was set in the substantia nigra (seed regions) to simulate the propagation process of MPs across the connectome. The results showed that the SIR model could effectively reproduce the spatiotemporal patterns of PD atrophy, with a significant correlation between real pattern and simulated pattern (*R* = 0.63). To further verify whether substantia nigra is the seed region for MP propagation, the researchers increased the initial concentration (1 × 10^−3^) of MP in 42 brain regions in sequence, with a step of 1 × 10^−3^. By using this method, the minimum concentration at which each brain region causes a large-scale outbreak of MP, namely the propagation threshold, can be found. They found that the propagation threshold was lowest in the substantia nigra, which might be the epidemic center of pathological protein spread in PD.

**Table 4:** Application of SIR model in neurodegenerative disease.

Literatures	Diseases	Subjects	Simulation data	Connectome	Seed	Correlation
Zheng and colleagues [18]	PD	237 PD, 118 HC	Cross-sectional T1w MRI, Resting-state functional MRI, AHBA	DSI	SN	*R* = 0.56
Rahayel and colleagues [60]	PD	87 mice	Longitudinal T1w MRI, Allen Mouse Brain Atlas API	Viral Tracking	Str, NAcc, HIP	*R* = 0.71, *R* = 0.77, *R* = 0.64
Abdelgawad and colleagues [61]	PD	790 PD, 279 HC	Longitudinal T1w MRIs, AHBA	DSI	SN	One year: *R* = 0.34, Two years: *R* = 0.33, Four years: *R* = 0.21
Shafiei and colleagues [62]	bvFTD	370 HC, 75 Genetic bvFTD, 70 Sporadic bvFTD	Cross-sectional T1w MRI, AHBA	DSI	Insula	*R* = 0.71
Rahayel and colleagues [63]	iRBD	261 HC, 182 iRBD	Cross-sectional T1w MRI, AHBA	DSI	STS	*R* = 0.52

The *R* in the table are all Pearson coefficients and represent the maximum values during the experimental process. The column “Simulation Data” and “Connectome” in the table respectively denote the data modalities utilized in the experiment and the data modalities employed for connectome generation.

PD, Parkinson’s disease; HC, healthy control; AHBA, Allen human brain atlas; T1w MRI, T1-weighted MRI; DSI, diffusion spectrum imaging; SN, substantia nigra; Str, striatum; NAcc, nucleus accumbens; HIP, hippocampus; bvFTD, behavioral variant frontotemporal dementia; iRBD, isolated rapid eye movement sleep behavior disorder; STS, superior temporal sulcus.

After demonstrating the power of the SIR model in simulating pathological protein spreading using cross-sectional data, the model also performed well in longitudinal simulations. Rahayel and colleagues [60] explored whether the SIR model could reconstruct the spatiotemporal patterns of pathological protein spreading in the PD mouse model. They selected 87 non-transgenic mice aged 2 to 3 months and injected α-syn preformed fibrils into their substantia nigra, ventral tegmental area, and hippocampus. The mice were followed for 2 years, and pathological α-syn content was measured at 7 time points. There was a significant correlation between the simulated and the real pathological protein spreading pattern (*R*_*max*_ = 0.77), and different injection points resulted in different pathological protein spread patterns [61–68]. Subsequently, the researchers verified the impact of gene expression and network connectivity on pathological α-syn spreading. Abdelgawad and colleagues [61] used a 4-year follow-up dataset (790 PD patients and 279 controls) to investigate whether the SIR model could reconstruct the longitudinal patterns of whole-brain atrophy in PD patients. The results indicated a significant correlation between observed data and simulated data at 3 different time points (1 year, *R* = 0.34; 2 years, *R* = 0.33; 4 years, *R* = 0.21), suggesting that the SIR model could reflect real pathological protein spreading process over time.

Meanwhile, the SIR model has also been applied to other neurodegenerative diseases. Rahayel and colleagues [63] used the SIR model to reconstruct the atrophy patterns in patients with rapid eye movement sleep behavior disorder. The simulated atrophy was significantly correlated with real atrophy (*R* = 0.52). Shafiei and colleagues [62] applied the SIR model to predict atrophy patterns in bvFTD. They first determined the epicenter as those regions with both severe atrophy and severe adjacent atrophy. Subsequently, they used the SIR model to validate the seed region without adding vulnerability information. The results indicated that the most likely seed region for bvFTD was the insula. Using the insula as the seed region, they simulated the evolution of atrophy throughout the entire brain using the SIR model, and the real atrophy pattern was significantly correlated with the simulated atrophy pattern (*R* = 0.6). Moreover, they incorporated gene expression distributions for *MAPT*, *GRN*, *C9orf72*, and *TARDBP* into the SIR model to characterize the production and clearance rates of pathological protein in different brain regions. The results showed that using the gene expression distributions of *C9orf72* and *TARDBP* yielded the best simulation, indicating that the expression of these genes affects the pathological protein spreading process of bvFTD. Altogether, the SIR model has potential applications in various central nervous system diseases, and more researchers may apply the SIR model to other neurodegenerative diseases.

To evaluate the importance of the selected features in simulating pathological spreading, Zheng and colleagues [18] utilized null models to explore the impact of connectome architecture (topology and geometry) on PD pathology. They established 2 types of null models for connectome architectures: (1) the topology of the connectome was randomized while preserving the original degree sequence and density; (2) the spatial positions of the regions were shuffled while preserving spatial autocorrelation, and the SIR models were then implemented on each null network and model fits were compared between the empirical and null networks. Their results implied that connectome topology and geometry jointly drive the high correspondence between simulated and observed atrophy in PD, with the agent-based SIR model showing significantly better fitting to empirical atrophy on the empirical structural network than that on either type of null network. Furthermore, a null model was also built for gene expression by shuffling expression profiles of *GBA* and *SNCA* across regions. Subsequent experiments revealed that the differential expression of genes across brain regions also influences the atrophy pattern in PD.

## Comparison of the biophysical models

The 3 biophysical models have distinct characteristics. We will primarily discuss the similarities and differences between the 3 models from 4 aspects. For detailed information, please refer to Table 5.

**Table 5:** Comparison of the 3 basic connectome-based biophysical models.

Model	NDM	ESM	SIR
**Diseases**	ALS, HD, COVID-19, FTD, TBI, PD, PSP, sCJD	AD	PD, bvFTD, iRBD
**Input**	Connectome, Real atrophy distribution vector, pathological protein distribution vector	Connectome, Pathological protein distribution vector	Connectome, Vulnerability vector, Real atrophy vector
**Simulation**	Pathology	Pathology	Pathology, Atrophy
**Research level**	Group/individual level	Group/individual level	Group/individual level
**Research perspectives**	Seed region and vulnerability	Seed regions	Seed regions and vulnerability
**Path of code**	https://github.com/Raj-Lab-UCSF	https://www.neuropm-lab.com/neuropm-box.html	https://github.com/yingqiuz/SIR_simulator

The diseases listed here are the ones already been reported. Moreover, the comparisons here are limited to the original models, the comparison of the improved models are not within the scope of discussion.

### Model complexity

To give a better understanding of the 3 base models, we compare their complexity here. Notably, the comparison only applies to the base models since the evolving extended models of the 3 base models have all presented a greater number of parameters to solve the potential pitfalls.

It can be seen from the mathematical principles that the NDM has only 1 unknown parameter, which is the propagation rate *β*. This parameter can be obtained through data-driven approaches, making the model relatively simple and convenient for integration with other mathematical models.

In contrast, from the formulas of the ESM, the ESM has 4 unknown parameters: the constant parameter infectious rate *β*_0_, the constant parameter clearance rate *δ*_0_, and the parameters *μ* and *σ* representing data noise. These 4 parameters need to be extracted through data-driven methods. Therefore, given that a large amount of real data is necessary for training the ESM model, it has a higher model complexity.

Regarding the SIR model, it has 3 free parameters *k*_1_, *k*_2_, and *ρ*_*i*_. The SIR model models brain atrophy as the result of 2 processes: (1) increasing toxicity within ROI leads to neuron death; (2) neuron death occurring in adjacent ROI causes the loss of neurotransmitters in neurons of this ROI, resulting in deafferentation and atrophy. *k*_1_ and *k*_2_ are the weights of the above 2 processes and satisfy that *k*_1_+*k*_2_ = 1. *ρ*_*i*_ represents the probability of pathological protein deposition in ROI_i_. Due to *k*_1_+*k*_2_ = 1, when $\frac{k_{1}}{k_{2}}$ changes between 0.1 and 10, the SIR model actually only has 2 free parameters, $\frac{k_{1}}{k_{2}}$ and *ρ*_*i*_.

### Can the epidemic center be identified?

A central tenet of the connectome-based spread hypothesis is that pathological proteins originate initially at one or more locations, often termed disease epicenters (true epicenter), and spread throughout the brain connectome, perhaps through synaptic contact [5]. However, the epicenter may also be certain brain regions adjacent to the true epicenter that exhibit high connectivity, efficient transmission pathways, or other features. These regions can be particularly effective at spreading the infection and may act as amplification nodes propagating the spread of pathological proteins throughout the network (propagator) [5]. From Table 5, it can be observed that all the 3 models require input vectors representing the distribution of pathological protein concentrations in different brain regions. Therefore, all 3 models can explore the optimal epidemic centers of neurodegenerative diseases by adjusting the seeded brain regions and concentrations. Taking NDM as an example, NDM can take an input vector *x*_0_ representing the initial pattern of the disease process.

When seeding at ROI_i,_ we only need to set the MP concentration in the corresponding ROI of the *x*_0_ vector to the target value. Therefore, NDM can seed in single/multiple brain regions while controlling the initial MP concentrations in all brain regions during seeding.

### Can the pathological proteins accumulation and brain atrophy pattern be predicted?

From their principles, all 3 models can predict the spreading patterns of pathological proteins. It is evident that the SIR models explicitly establish mapping functions between changes in pathological proteins and brain atrophy. Therefore, SIR can predict brain atrophy. However, further validation and model optimization is necessary before linking the simulation results of NDM and ESM directly to brain atrophy.

### Is it possible to study regional vulnerability?

Due to variations in the local properties of the brain, specific brain regions are more vulnerable to the deposition of pathological proteins compared to others. The spreading of pathological proteins is not only constrained by the brain connectome but is also regulated by various vulnerability factors. Common vulnerability factors include regional gene expression, local metabolic levels (PET, arterial spin labeling, blood oxygen level-dependent functional magnetic resonance imaging), local immune levels (diffusion-weighted imaging or PET), and other parameters that represent local characteristics of brain regions. Among the 3 models, NDM and SIR can be more flexibly combined with vulnerability mechanisms, allowing for exploring the impact of different vulnerability factors on the spreading. For example, one extended NDM approach incorporated parameters characterizing spatial gene expression-dependent intra-regional accumulation and inter-regional spreading. They found that the NDM incorporating baseline expression of *TREM2* outperformed the model without gene expression information [69]. Meanwhile, ESM encapsulates its code into a software tool, making it less straightforward to add additional parameters. Furthermore, ESM has a higher model complexity and performs well but carries a risk of overfitting. It is essential to reach a balance between improving model performance and avoiding overfitting when adding other parameters.

## Model optimization

While the 3 biophysical models have made significant progress in the study of neurodegenerative diseases, there is still potential for further optimization. Optimization can be achieved from multiple aspects.

### Setting the direction of propagation

All 3 biophysical models do not specify the direction of pathological protein spreading, but in actual physiological processes, the propagation of pathological proteins within neural networks exhibits directionality.

Some studies suggest that the simulation with a directed structural connectivity network yields better results than using an undirected structural connectivity network [23]. When optimizing models, parameters can be further introduced to control the direction of propagation of pathological protein between different ROIs. Furthermore, the next-generation high-resolution diffusion-weighted imaging techniques, which could reflect directed structural connectivity networks, are expected to enhance model performance.

### Enhancing the validity of model parameters

All the NDM, ESM, and SIR models simplify complex biological processes during modeling. In the NDM model, during the initial seeding stage, the MP diffuse from the seed region to other regions under the influence of concentration differences between ROIs. However, evidence has found that some highly connected regions (e.g., striatum) of probable seed (e.g., SNc) do not show pathology, which indicates that simple network diffusion is not the best model to describe this dynamic process. Some recent advances proposed that the reaction-diffusion model could be more accurate and flexible compared to the simple diffusion model [31]. Different types of disease progression could be modeled by specifying different reaction terms and initial conditions for the reaction-diffusion model, which holds the potential to serve as a unified framework for simulating the progression of various neurogenerative diseases. Future investigations could examine the predictive performance of the reaction-diffusion model in various diseases, as well as improve the model by introducing non-constant system parameters that allow different diffusion rates in different regions, which could be a great extent to the modeling of disease progression.

Additionally, the mapping relationships between diffusion speed and concentration differences of pathological proteins, as well as between brain atrophy and pathological protein deposition, are relatively simplistic and cannot fully reflect the actual diffusion process. In the SIR model, the model simplifies the propagation rate between edge-ROI pairs as the inverse of fiber length, specifies production and clearance rates as cumulative distribution functions of gene expression distribution, and models atrophy as a linear combination of local neuron death and deafferentation. These assumptions may not fully reflect the actual propagation process. To reduce the gap between simulated and real progression of neurodegenerative diseases, the following approaches can be considered: (1) Incorporate more biological details and mechanisms into the models to simulate pathological spread processes more accurately. (2) Add more clinical predictive factors, such as genes, age of onset, and education level, to increase the complexity of the model and better capture the diversity and complexity of biological systems.

Moreover, although significant model advancements have been observed over the years, many of the advanced models have not been quantitatively examined in clinical settings, i.e., comparing simulated results with observed patient data. Future examination of these advanced models in patients could pave the way to an in-depth understanding of the mechanism of diseases and successful clinical translation.

### Improving the correspondence of the time scale

Among the 3 models, only the simulated pathological protein spreading process of the ESM model is aligned with actual clinical time. In contrast, the simulation processes of the NDM and SIR models cannot accurately correspond to the actual clinical age of onset and follow-up time. Future studies could introduce time-related parameters into the models to ensure alignment between actual time and simulated time, increasing the validity of the models.

### Interdisciplinary communication

Future research could benefit from collaboration with experts from fields such as neuroscience, biology, and medicine, who can update the knowledge about disease mechanisms and biological processes. This knowledge can be introduced into the model structures or parameter settings and better capture the actual dynamic processes of pathological protein propagation. Moreover, the SIR and ESM models have drawn inspiration from ideas and methods in population-level epidemiology of infectious diseases. The fruitful research outcomes and ongoing advancements in the field of epidemiology will continue to offer valuable insights and methods for optimizing models of pathological protein propagation in neurodegenerative diseases.

## Individualized simulation

For individualized measurements of real deposition or atrophy, PET measurements of pathological deposition can reflect the individual-level deposition location and severity of pathological proteins. However, since brain atrophy needs to be obtained by comparing with normal individuals, many previous studies have used between-group volume differences to measure real atrophy. To obtain individual-level measurements of real atrophy, researchers often use normative models to compare individual gray matter structural attributes with predefined populations, calculating the differences of gray matter structural attributes between the individual and normal population with equivalent attributes (age, gender, and education level) [63].

However, the studies using individualized measurements of real atrophy or deposition typically employ group-level structural connectivity and vulnerability factors, which leads to a lack of individual specificity and may not meet the demands of clinical practice. To improve the clinical applicability of these models, future research could explore the impact of individual-level structural connectivity and vulnerability factors on simulation performance. The individual structural connectivity network obtained with current diffusion-weighted imaging, model reconstruction, and tracking technologies is feasible, and the robustness and stability of individual structural connectivity networks from previous research are generally adequate for simulation [70–72]. It should be noted that the current propagation of pathological protein models typically uses structural connectivity data from normal subjects for propagation. Future research needs to compare the simulation performance using the structural connectivity in disease and normal states to determine if normal structural connectivity is necessary.

**Fig 1:**
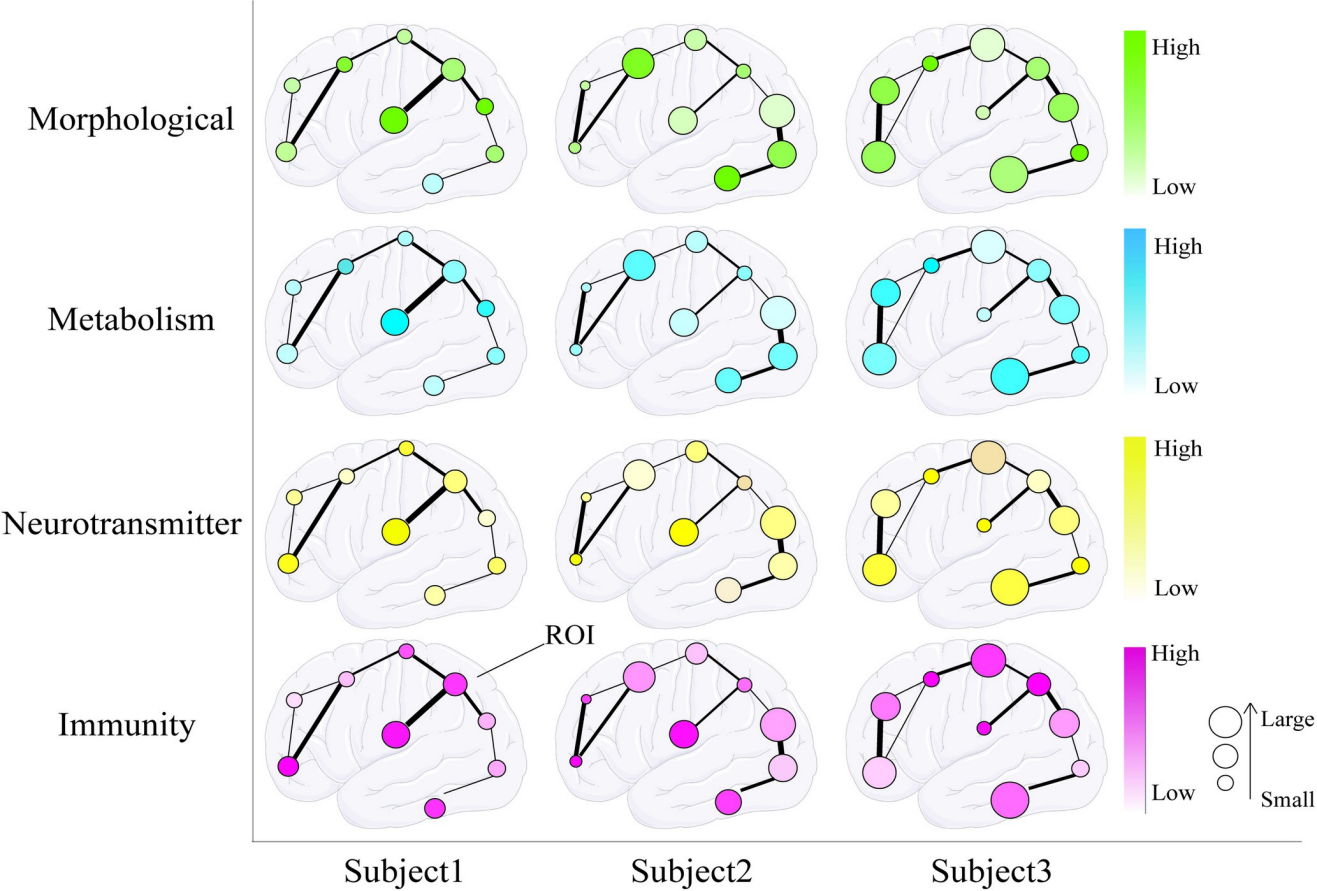
Individual difference in connectivity pattern and vulnerability mechanisms. The horizontal axis represents individual difference in the ROI size and location, along with the difference in structural connectivity patterns. The vertical axis represents the distribution of vulnerability factors within the brain of the same individual, including morphological attributes (gray matter, white matter/myelin sheath, synaptic density, etc.), energy metabolism (glucose, blood flow, aerobic glycolysis, oxygen saturation of blood, etc.), neurotransmitter systems (receptors, transporters), and immunity (activated microglia, activated astrocytes). The distribution of vulnerability factors vary across different ROIs within the same individual. In the brain graph, circles represent ROIs, line represent structural connections between ROIs. The size of the circles indicates the size of the ROIs, the color from light to dark represents the distribution of vulnerability factors across different ROIs and the thickness of the lines represents the strength of structural connections. The brain of left hemisphere sketch in Fig 1 is downloaded from the open-source site Servier (URL: https://smart.servier.com/smart_image/brain/), which is licensed under CC BY 4.0.

Due to the particularity of clinical work and the requirements of the models, the measurements of individual-level vulnerability must be noninvasive, harmless, and have high spatial resolution. Therefore, measuring individual-level local gene expression and cell type distributions is quite challenging. The individual-level local vulnerability factors that can be measured with noninvasive imaging techniques include morphological structure [73] (gray matter [74], white matter/myelin [75–78], synaptic density [79,80], etc.), energy metabolism (glucose [81], blood flow [82,83], aerobic glycolysis [84,85], oxygen saturation of blood [86,87], etc.), neurotransmitter systems [88] (receptors, transporters), and immune system (activated microglia [89], activated astrocytes [90], etc.), among other vulnerability factors. Fig 1 illustrates the individual differences in connectivity patterns and vulnerability factors.

## Unified model evaluation framework

**Fig 2:**
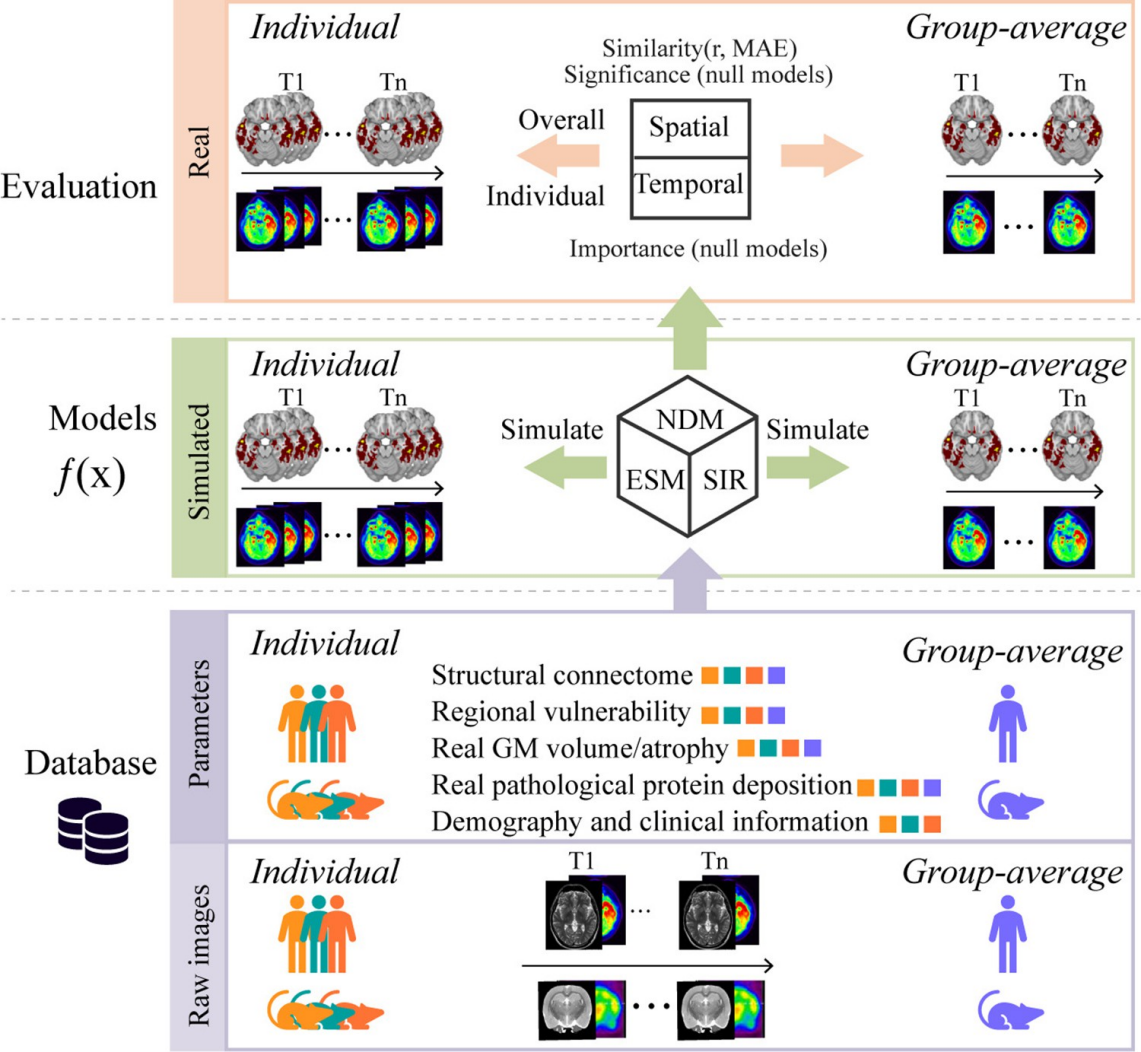
The unified framework for model evaluation. **Database:** The unified database includes both raw data and parameter libraries, which are divided into individual-level data and population-level data, animal and human brain images, cross-sectional data and longitudinal data with multiple time points. **Models:** These data are used for model *f*(*x*), where researchers select appropriate biophysical models (NDM, ESM, and SIR) for individual-level or population-level analysis as appropriate. The selected model was fitted using the data provided by the parameter library and the simulated propagation process of pathological proteins/brain atrophy was obtained. **Evaluation:** The simulation results are compared with the real propagation process of pathological proteins/brain atrophy from both temporal and spatial perspective, utilizing measures of model performance (e.g., Pearson’s r, MAE), significance (spatial autocorrelation-preserving null models), and importance of model parameters (null models). The human and mouse icons are free icons provided by Microsoft PowerPoint.

The 3 biophysical models have demonstrated satisfactory performance in simulating the process of neurodegenerative disease progression. However, they share common limitations. Researchers have evaluated the models using different data sources without a unified dataset and consistent standards for evaluating model performance. These issues make it challenging to conduct quantitative comparisons between models or highlight the specific characteristics of a single model. To address these issues, we propose a unified framework with unified dataset and evaluation method that will significantly impact model parameter selection, optimization, and performance evaluation. Fig 2 illustrates the entire evaluation framework.

First, a unified dataset is established. This dataset includes individual-level and population-level, animal and human, cross-sectional and longitudinal neuroimaging data. For each individual, the dataset contains a comprehensive set of parameters, such as individualized brain segmentation (brain region localization and size), structural connectivity, vulnerability factors, pathological protein distribution, atrophy distribution, cognitive scores, age, gender, and education level. For a group of subjects, the dataset contains group-level structural connectivity, pathological protein distribution, and atrophy distribution. Using the same dataset, researchers can choose the appropriate biophysical model (NDM, ESM, SIR, etc.) for population-level or individual-level analysis, determine the parameter values using the parameter library, and then input custom information to simulate the spreading of pathological proteins or brain atrophy.

Following this, it is essential to evaluate the performance of the model. Previous studies have primarily evaluated model performance in terms of spatial similarity using the following assessment methods: (1) calculating the Pearson or Spearman correlation coefficient between the real data and the simulated data at a given time point [8,15,18,60,61]; (2) calculating mean absolute error (MAE), the error here refers to the difference between the real data and the simulated data for each brain region. MAE is obtained by summing all brain regions’ absolute errors and dividing by the number of regions. The smaller the MAE, the more similar the simulated data is to the real data [8]; (3) performing cross-validation to reduce the potential overfitting problem of the biophysical models, which divides the participants into the training set and the test set. The connectome-based biophysical models are optimized using the training set and evaluated on the test set. By comparing the simulated data with the real data on the test set, the average of multiple measures of fit (e.g., MAE, EVS) can be used as indicators of simulation performance [8,15,22,24,52]. However, limited attempts have been made to evaluate models from a temporal perspective. The development and propagation of neurodegenerative diseases are dynamic processes, so improving the alignment of actual time and simulation time can better reflect important information that changes over time, such as the spreading of pathological proteins and brain morphometric change. Accordingly, future model performance evaluations need to consider both temporal and spatial dimensions. In other words, simulated data should exhibit high spatial similarity to real data not only at a single time point but also across various time points.

To assess the significance of biophysical model performance, null models have emerged as a prevailing method for generating control parameters that preserve specific features of the parameter while randomizing other features. They are extensively used to evaluate the statistical significance and meaningfulness of observed model parameter [91,92]. Previous studies (e.g., Zheng and colleagues [18]) have employed null models to directly estimate the significance of gene expression and connectome as a specific model parameter of interest. For example, by simulating the spreading of pathological proteins with randomized gene expression profiles or rewired connectome (topology or geometry), these null models help to establish the importance of empirical gene expression and connectivity pattern in the pathological spreading of neurodegenerative disorders [60–63]. Another important consideration when comparing simulation result to real data is the need to account for spatial autocorrelation in brain data. As an alternative to extensively applied parametric testing, Markello and colleagues [91] conducted a comprehensive evaluation of the performance of 10 published null models in statistical analysis of neuroimaging data and found that null models that preserve the spatial autocorrelation of brain maps yielded more accurate statistical estimates. Therefore, in future studies, it is recommended to apply null models in 2 ways: one is to simulate with model parameters generated by null models to evaluate the importance of specific parameters of interest (e.g., connectome or expression), and the other one is to utilize spatial autocorrelation-preserving null models when assessing the significance of similarity between empirical and simulated data. Similar to the measure of model performance, the significance of the model performance and the importance of model parameters are also encouraged to be measured in both temporal and spatial dimensions.

Furthermore, with a consistent database and evaluation framework in place, organizing open algorithm competitions and releasing competition tasks can accelerate data accumulation and model optimization, promote the integration of interdisciplinary methods, and advance the clinical application of connectome-based biophysical models.

## Conclusions

In summary, the development and application of NDM, ESM, and SIR models have made significant contributions to unraveling the progression of neurodegenerative diseases. This review has introduced the basic principles and applications of 3 biophysical models, compared their similarities and differences, and proposed directions for the development of next-generation connectome-based biophysical propagation models (model optimization and personalization). Furthermore, this review suggests establishing a unified model evaluation framework, including open benchmark datasets and spatiotemporal similarity evaluation metrics. This review could benefit the field from 2 aspects: First, we provide researchers with guidelines for selecting suitable biophysical models according to their research interests. Second, it allows for the objective and quantitative comparison of the simulation performance of different biophysical models, which helps to standardize the development and usage of biophysical models in this field and accelerate model optimization in various scenarios.
